# Draft Genome Sequence of Persistent Klebsiella grimontii AT013-Mero-001, Isolated from Human Feces

**DOI:** 10.1128/MRA.00054-21

**Published:** 2021-04-22

**Authors:** Sarah Bühler, Jürgen Rödel, Bettina Löffler, Michael Bauer, Anne Busch

**Affiliations:** a Department of Anaesthesiology and Intensive Care Medicine, University Hospital Jena, Jena, Germany; b Department of Medical Microbiology, University Hospital Jena, Jena, Germany; University of Maryland School of Medicine

## Abstract

Here, we report the draft genome sequence of Klebsiella grimontii AT013-Mero-001, which was isolated from feces from a sepsis patient treated with meropenem. This isolate is an antibiotic-susceptible but persistent *Enterobacteriaceae* strain.

## ANNOUNCEMENT

*Klebsiella* spp. are known for hospital-acquired infections (1–3). *Klebsiella* is known to be able to survive by persisting following long-term starvation (4) and to develop tolerance in the presence of an antibiotic without an increase in the MIC (5). Here, we present a draft genome sequence of Klebsiella grimontii AT013-Mero-001, which was isolated from feces from a sepsis patient who was treated with meropenem in the University Hospital Jena (Jena, Germany) in 2019. It was isolated on MacConkey agar. Although the strain was initially identified as Klebsiella oxytoca by matrix-assisted laser desorption ionization–time of flight mass spectrometry (MALDI-TOF MS) as described previously (6), 16S RNA extraction from the assembly by mapping with Geneious (7), analysis with MOLE-BLAST (8, 9), and average nucleotide identity analysis (10) identified the strain as Klebsiella grimontii. Default parameters were used for all software unless otherwise specified.

Antimicrobial susceptibility was assessed by determining the clinical MICs, in accordance with the EUCAST breakpoints (11), using the Vitek Compact system (bioMérieux, Marcy-l'Étoile, France). The strain was categorized as resistant to ampicillin (MIC of 18 mg/liter), susceptible to ampicillin-sulbactam (MIC ≤ 2 mg/liter), resistant to piperacillin (MIC ≤ 4 mg/liter), susceptible to piperacillin-tazobactam (MIC ≥ 4 mg/liter) (Advanced Expert System [AES] modified), susceptible to cefuroxime (MIC ≤ 2 mg/liter), susceptible to cefuroxime-axetil (MIC ≤ 1 mg/liter), susceptible to ceftazidime (MIC ≤ 1 mg/liter), susceptible to ertapenem (MIC ≤ 0.5 mg/liter), susceptible to imipenem (MIC ≤ 0.25 mg/liter), susceptible to meropenem (MIC ≤ 0.25 mg/liter), susceptible to gentamicin (MIC ≤ 1 mg/liter), susceptible to ciprofloxacin (MIC ≤ 0.25 mg/liter), susceptible to tigecycline (MIC ≤ 0.5 mg/liter), and susceptible to trimethoprim-sulfamethoxazole (MIC ≤ 20 mg/liter).

The strain was grown on Columbia blood agar with 5% sheep blood (Becton, Dickinson GmbH, Heidelberg, Germany). DNA from whole genomes was prepared with the Nextera DNA Flex microbial colony extraction protocol (12) and the Nextera Flex DNA preparation kit (13) as the manufacturer instructed. Paired-end sequencing was performed on the MiSeq platform (Illumina, Inc., San Diego, CA, USA) using a 300-cycle MiSeq reagent kit with a read length of 151 bp. A quality check was performed with FastQC v0.11.9. A total of 1,212,398 reads were *de novo* assembled using SPAdes v3.14.1 in BayesHammer mode (--careful) (14), evaluated with QUAST v4.3 (15, 16), and filtered as described previously (17). ResFinder v4.1 (18) predicted acquired antimicrobial resistance to ampicillin (*bla*_OXY-6-2_ [GenBank accession number AJ871875]) and mutations in genes known for ampicillin resistance; although seven mutations were detected in the *ompK36* gene (p.A183S, p.F198Y, p.L229V, a190_None191insW, p.F207Y, p.N304E, and p.E232R), no carbapenem resistance was observed. No plasmids were detected in agarose gels or with PlasmidFinder v2.1 (19). The strain was identified as sequence type 261 using multilocus sequence typing (MLST) v2.0 based on next-generation sequencing (NGS) data (20).

The genome assembly was represented by 114 contigs with a contig *N*_50_ value of 91,957 bp; the largest contig was 319,034 bp. The average coverage was >90-fold. The combined length was 5,831,782 bp, with a G+C content of 55.7%. Annotation was performed with PGAP (21–23). Annotation features include 5,713 DNA coding sequences (CDSs), 6 rRNAs, 2 repeat regions, 64 tRNAs, and 1 transfer-messenger RNA. These data are in concordance with those for other *Klebsiella* strains (24–26). The ATHENS study was approved by the local ethics committee in Jena, Germany (protocol 5289-10/17). Written informed consent was obtained from all patients or their legal representatives.

### Data availability.

The whole-genome sequence of Klebsiella grimontii AT013-Mero-001 was submitted to NCBI under BioProject number PRJNA690555 and BioSample number SAMN17250868, with SRA accession number SRP301190 and GenBank accession number JAFBAX000000000. The version described in this paper is the first version.
